# Current Evidence of the Effect of Breastfeeding on Ear Molding Outcomes: A Scoping Review

**DOI:** 10.1002/lary.70413

**Published:** 2026-02-06

**Authors:** Harry Chiang, Samuel R. Shing, Peggy Su‐Genyk, Lilun Li, Kelvin Kwong, Joseph B. Vella

**Affiliations:** ^1^ Glasgold Group Plastic Surgery Princeton New Jersey USA; ^2^ Department of Head and Neck Surgery & Communication Sciences Rutgers Robert Wood Johnson Medical School New Brunswick New Jersey USA

**Keywords:** auricular deformity, auricular molding, ear molding, microtia, otoplasty

## Abstract

**Objective:**

To provide an overview on auricular molding and to investigate whether breastfeeding is associated with greater cartilage malleability or improved perinatal auricular molding outcomes.

**Data Sources:**

Embase, MEDLINE, and CENTRAL databases.

**Methods:**

Three databases were comprehensively searched for treatment of congenital auricular malformations or deformations with auricular molding. Extracted data included study design, patient demographics and auricular anomalies, interventions and outcomes measures, and references to the effect of estrogen and breastfeeding on cartilage malleability and ear molding.

**Results:**

Out of 1018 unique articles, 67 were included in this review. The articles included a total of 3645 patients and 5384 ears, of which helical rim abnormality (20.6%), lop/lidding/cup ear (20.2%), and prominent ear (11.1%) were the most represented. Of the 67 articles referencing estrogen levels, 49 supported the claim that estrogen increases auricular cartilage malleability. Fifteen articles (22.4%) referenced the effect of breastfeeding on infant circulating estrogen levels and molding outcomes, while 8 (53.3%) of those articles supported the claim. Notably, only 4 primary sources were referenced regarding the effect of breastfeeding among all reviewed literature.

**Conclusions:**

There is insufficient evidence to suggest that breastfeeding leads to increased circulating estrogen levels in infants, increased auricular cartilage malleability, or improved ear molding outcomes. The direct impact of hyaluronic acid on the mechanical properties of auricular cartilage in vivo remains unclear.

## 1 Introduction

Congenital auricular anomalies affect social interaction in infancy as self‐awareness develops [1, 2]. Embryologic development of the auricle begins during week five of gestation with coordinated fusion of six mesenchymal hillocks from the first and second branchial arches, and the auricular cartilaginous framework is developed by week nine [3, 4]. The auricular contour continues to change, as the auricle undergoes various forces from muscle insertions and external forces both in utero and postnatally [3]. Disruption in development prior to week nine of gestation may lead to auricular malformations, where components of the cartilaginous framework may be absent or duplicated (e.g., microtia, anotia). Disruption of auricular maturation after week nine results in deformations, where the framework is present, but distorted (e.g., lop or cup ear, Stahl's ear, prominent ear, helical and conchal abnormalities) [5]. While most ear malformations require surgery, ear deformations can successfully be treated with non‐surgical techniques to restore contour.

Auricular molding has become an increasingly popular non‐surgical option for treating auricular deformations and select cases of mild malformations. Ear cartilage is an elastic cartilage consisting of chondrocytes surrounded by an extracellular matrix of collagen, elastin, and proteoglycans [6, 7, 8, 9]. In the postnatal period, estrogen is theorized to lead to increased levels of hyaluronic acid (HA)—a constituent of proteoglycan aggregates—which creates a water‐rich environment within the auricular cartilage to increase malleability. However, circulating neonatal estrogen levels are highest at birth and rapidly decline within 72 h, with a 50% decrease within 30 h [10]. Auricular molding is thought to target this early window for introducing biologic creep by applying prolonged stress to the cartilage and triggering tissue remodeling [11, 12].

While the effect of estrogen on HA and cartilage malleability has deeply permeated the literature, the mechanism of this process is poorly described. Although animal studies have reported that intravenous estrogen injection increases HA content, this effect was seen in arteriole walls and skin, but not clearly in cartilage [13, 14, 15]. The proposed mechanism of circulating estrogen levels increasing HA and thus auricular malleability appears mostly speculative, based on temporal associations between diminishing estrogen after birth and diminishing success with delayed initiation of molding. While frequently cited studies recommend breastfeeding as a way to extend the window for auricular molding by increasing neonatal estrogen levels during the nursing period, this recommendation deserves further assessment based on limited evidence [5, 16]. In fact, a recent review by Jin et al. found no evidence that breastfeeding infants demonstrate signs of elevated circulating estrogen [17].

The purpose of this scoping review is to provide an overview on auricular molding and to query the literature for evidence on the impact of breastfeeding on estrogen levels and auricular molding outcomes in infants. Specifically, in infants undergoing auricular molding for auricular deformations, is breastfeeding associated with greater auricular cartilage malleability through an estrogen‐mediated increase of HA? By critically analyzing the literature, we aim to identify the primary evidence supporting or refuting these claims, identify gaps in knowledge regarding the mechanisms by which HA may increase auricular malleability, and provide directions for future study.

## 2 Methods

A scoping review was reported in accordance with the Preferred Reporting Items for Systematic reviews and Meta‐Analyses extension for Scoping Reviews checklist [18]. The Embase (Elsevier), MEDLINE (National Library of Medicine), and CENTRAL (Wiley) databases were searched on August 30th, 2025 to identify publications investigating ear molding for auricular malformation or deformation with no limit on date. The search used keywords and Boolean operators as follows: (“ear” OR “auricle” OR “auricular”) AND (“deformity” OR “abnormality” OR “malformation” OR “microtia”) AND (“mold” OR “molding” OR “splint” OR “splinting” OR “nonsurgical” OR “noninvasive”), as shown in Supporting Information Table 1. Identified studies were imported into Covidence Systematic Review Software (Veritas Health Innovation, Melbourne, Australia). Duplicates were removed, and two authors (HC, SRS) independently screened articles for inclusion, with conflicts settled by consensus. Inclusion criteria were English full‐texts focused on ear molding for auricular deformation or malformation with mentions of the effect of estrogen and/or breastfeeding. Exclusion criteria were single case reports, communications, studies focusing on surgical technique or prosthetics, studies focusing on aging or acquired earlobe deformity, studies not referencing the effect of estrogen and/or breastfeeding, and exclusively animal studies.

Extracted data included journal specialty, study design, sample size, demographics, anomaly type, interventions and outcome, mentions of the impact of estrogen or breastfeeding on ear molding, and citation patterns regarding the effect of estrogen and breastfeeding on ear molding. A recent review of auricular molding by the senior author had noted seven primary sources that were heavily referenced [19]. Particular attention was given to any references to these sources [10, 13, 14, 15, 20, 21, 22]. Details about complications of auricular molding and specific metrics for outcome assessment after auricular molding have been summarized in several recent reviews and were considered beyond the scope of this paper [23, 24, 25, 26, 27].

## 3 Results

### 3.1 Study Selection and Patient Demographics

**FIGURE 1:**
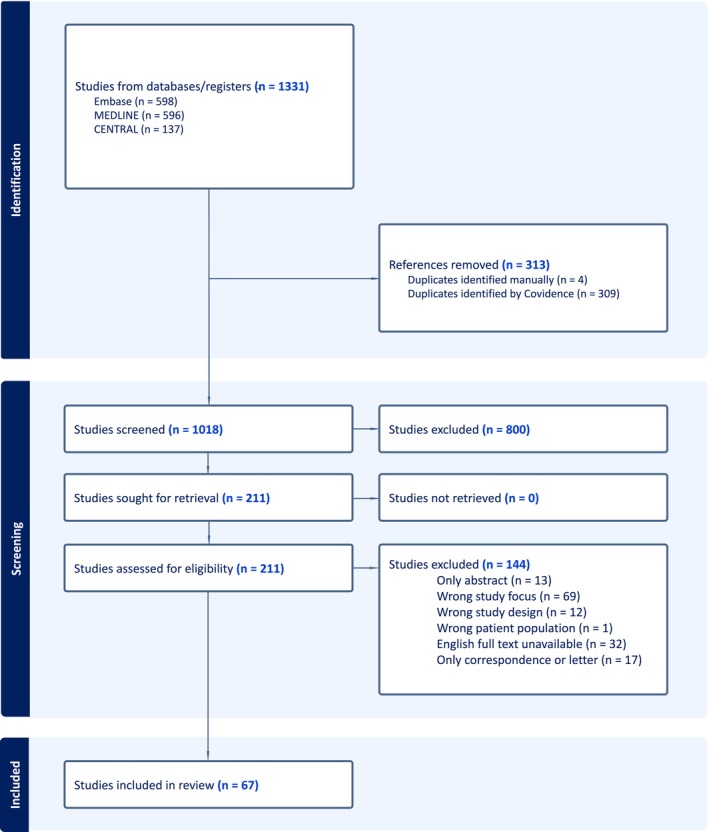
PRISMA diagram of search strategy. [Color figure can be viewed in the online issue, which is available at www.laryngoscope.com]

The search identified 1018 unique articles regarding ear molding for auricular malformation or deformation. Eight hundred articles were excluded after title and abstract screening, and 211 full‐text articles were reviewed for inclusion. Sixty‐seven articles were included in the review (Figure 1) [5, 12, 16, 19, 23, 24, 25, 27, 28, 29, 30, 31, 32, 33, 34, 35, 36, 37, 38, 39, 40, 41, 42, 43, 44, 45, 46, 47, 48, 49, 50, 51, 52, 53, 54, 55, 56, 57, 58, 59, 60, 61, 62, 63, 64, 65, 66, 67, 68, 69, 70, 71, 72, 73, 74, 75, 76, 77, 78, 79, 80, 81, 82, 83, 84, 85, 86].

The majority of studies were retrospective case series (*n* = 33), followed by prospective case series (*n* = 12), literature reviews (*n* = 13), systematic reviews (*n* = 7), and other (*n* = 2). Thirty articles were published in plastic and reconstructive surgery journals, followed by 17 in otolaryngology or facial plastic surgery, 8 in pediatrics, 7 in craniofacial, and 5 in other specialty journals. Excluding the patients described in systematic reviews, a total of 3645 patients and 5384 ears were described in the articles. The auricular anomalies included helical rim abnormalities [*n* = 1110 (20.6%)], lop/lidding/cup ear [*n* = 1087 (20.2%)], prominent ear [*n* = 598 (11.1%)], Stahl's ear [*n* = 355 (6.6%)], cryptotia [*n* = 316 (5.9%)], and conchal abnormalities [*n* = 103 (1.9%)]. Three hundred and twenty‐two (6.0%) ears were described as mixed deformities. The remaining ears belonged to other categories of deformation or were unspecified (Supporting Information Table 2). Four studies included microtia (22 ears). To the authors' knowledge (excluding systematic reviews), no two articles had identical patient series, although overlapping cases across studies could not be excluded.

### 3.2 Auricular Molding Variations and Outcome Assessment

Most studies used commercial auricular molding systems (*n* = 32), including EarWell (Becon Medical, Batavia, IL) (*n* = 25) and InfantEar (TalexMedical, Malvern, PA) (*n* = 5). Overall, there was similarly high family satisfaction after auricular molding with EarWell and InfantEar [78]. However, reported limitations of the EarWell and InfantEar systems include high cost and lack of customizability. As such, there has been interest in lower‐cost commercial systems such as LiangEar (Jiangsu Deviceland Medical Instrument Corp., China) and GTK Ear Correction (Guangzhou T.K Medical Instrument Co., China), as well as custom constructs using alternative materials to offer more customization that addresses the variable nature of auricular anomalies [45, 50, 79, 87].

Age at treatment initiation varied widely. Among studies that presented mean age at treatment initiation, mean age ranged from less than 1 week to approximately 14 weeks (3.5 months), although most studies initiated treatment within 3–4 weeks. There was a general consensus that earlier initiation was associated with improved outcome [12, 16, 39, 45, 56], although there was no consistent window identified across studies. Some advocated for initiation prior to 1–3 weeks, while others reported success beyond 6 months [16, 56, 75, 88, 89, 90]. There was no increase in complications with delayed initiation, although there was a decreased likelihood of success [56]. There was little consensus regarding duration of treatment, which varied from a mean duration of 2–3 weeks up to approximately 22 weeks (5.5 months). In many studies, duration of treatment depended on clinical progress, and molding continued until a positive outcome was achieved or treatment was aborted due to complications.

### 3.3 Circulating Estrogen and Auricular Malleability

Of 67 studies referencing the effect of estrogen on auricular cartilage malleability, 49 (73.1%) supported the claim that increased circulating estrogen increases cartilage HA and auricular malleability, 17 (25.4%) were neutral, and 1 (1.5%) rejected the claim. Thirty‐two of the 67 articles (47.8%) cited a primary source regarding the effect of estrogen, and of these, 28 of 32 articles (87.5%) cited at least one of the 7 articles of interest noted by the senior author [10, 13, 14, 15, 20, 21, 22].

### 3.4 Breastfeeding Effect on Estrogen Levels

Fifteen articles (22.4%) referenced the impact of breastfeeding on infant circulating estrogen. Of these, eight articles (53.3%) supported the claim that breastfeeding increases circulating estrogen (or that breastfeeding is associated with increased cartilage malleability) while three articles (20%) were neutral, and four articles (26.7%) rejected the claim. Of the 15 articles referencing breastfeeding, 10 articles either cited primary sources or were primary studies. Among the 10 articles, 4 (40%) supported the claim while 6 articles (60%) were neutral or rejected the claim.

On close inspection of the cited sources describing the effect of breastfeeding on auricular molding outcomes, there were only four primary studies. One study anecdotally described increased auricular malleability in breastfeeding patients, and three studies provided quantitative analyses of the impact of breastfeeding on ear molding outcome [5, 33, 45, 79]. The most commonly cited study was by Tan et al., which anecdotally reported increased pliability of ear cartilage with breastfeeding across 32 ear deformities; there was no control group or quantitative analysis [5]. In a prospective study over 3 years of 105 ear deformations, Chan et al. found no difference in outcome between infants that did or did not breastfeed [33]. Similarly, Hui et al. found no difference in outcome among 59 ears that underwent auricular molding between breastfed, mixed, or formula‐fed infants [45]. Xiong et al. conducted a case series of 522 ears and found that concurrent breastfeeding, along with earlier treatment initiation, longer treatment duration, and milder auricular deformation were all associated with significantly improved outcomes after multivariate analysis [79]. There is equivocal support for breastfeeding improving auricular molding outcomes and insufficient evidence to provide a recommendation regarding breastfeeding in patients undergoing auricular molding.

## 4 Discussion

Auricular molding has been in practice since the 1960–1970s, with most reports dating back to the 1980s [12, 31, 67, 91, 92]. However, there remains limited understanding of the optimal timing, duration, and technique of treatment for a pathology that encompasses a wide breadth of presentations. In particular, breastfeeding is often recommended in clinical practice as a way to improve auricular molding outcomes. Commonly cited studies propose a physiologic pathway of breastfeeding increasing circulating estrogen levels, thereby increasing HA levels and auricular cartilage malleability. However, the direct association between breastfeeding, estrogen, and auricular cartilage malleability hinges upon conflicting reports along the proposed pathway. This scoping review aimed to investigate the claim that breastfeeding in infancy is associated with greater auricular cartilage malleability through an estrogen‐mediated increase in HA. To our knowledge, this review encompasses the largest aggregate of studies regarding auricular molding to date.

A recent review by the senior author identified seven articles that constituted the majority of the basis for associating circulating estrogen with auricular malleability in the literature. As such, close inspection of these articles is warranted. In 1961, Schiff and Burn found a qualitative increase in acid mucopolysaccharides in cheek pouch biopsies of hamsters, monkeys, and humans, after intravenous estrogen injection [15]. This effect was observed primarily near small blood vessels. In 1972, Hardingham and Muir found that the addition of HA to porcine laryngeal cartilage increased the hydrodynamic size and viscosity of proteoglycan aggregates [21]. In contrast, Hascall and Heinegard found that HA decreased the viscosity of proteoglycan aggregates in bovine nasal cartilage [20]. In 1973, Kenny et al. reported that upon birth, there is a rapid decline in human neonatal unconjugated estrogen levels within the first 30–38 h, followed by a decline of two orders of magnitude at 72 h [10]. Similarly, Hung wrote in 1978 that neonatal estrogen levels are higher in the first week of life, although the full‐text was unavailable [22]. In 1980 and 1981, Uzuka et al. found that in mouse dermis, there was a dose‐dependent increase in HA through upregulation of hyaluronic synthetase with subcutaneous injection or topical administration of estradiol, along with an associated increase in water content [14, 15]. Taken altogether, estrogen may increase HA in dermis and near blood vessels, but its effect on HA in cartilage remains unclear.

More recent studies have shown that estrogen receptors are present in human skin and cartilage [93, 94, 95]. In cartilage, estrogen is theorized to regulate chondrocyte proliferation and maturation, proteoglycan synthesis, and secondarily, HA production and collagen synthesis [95]. In turn, increased HA may lead to water retention, thus changing the mechanical properties of the cartilage. However, a critical review of the existing literature shows that while estrogen may increase HA levels in dermis and near blood vessels through upregulation of hyaluronic synthetase, this association has not been shown in cartilage. Two studies investigating auricular molding in rabbit ears in vivo found that rabbit ears injected with subcutaneous estrogen or treated with topical estrogen demonstrated improved modification of rabbit ear angles, but there was no difference in HA expression in the ear cartilage itself [96, 97]. Rather, hyaluronic acid expression was increased in splinted compared to unsplinted ear cartilage, not estrogen treated versus untreated controls, suggesting that mechanical splinting itself may play a role in hyaluronic acid levels [96]. In addition, there are no studies that show increased hyaluronic acid expression in human cartilage after estrogen treatment.

There is a large breadth of research on the mechanical properties of auricular cartilage in the tissue engineering domain that is poorly represented in the ear molding literature [8, 98, 99]. While the role of HA in ear molding is commonly described, the role of elastin is not well‐studied. Auricular cartilage contains higher levels of elastin compared to hyaline cartilage. Massengill et al. demonstrated that elastin, similarly to HA, contributes to the mechanical properties of auricular cartilage [100]. In a rabbit model, two experimental groups (one with local injection of hyaluronidase and one with injection of elastase), followed by 4 weeks of splinting, led to comparable changes in malleability compared to the control [100]. Another study suggested that elastin may be more responsible for the mechanical properties of auricular cartilage than glycosaminoglycans [98]. In bovine auricular cartilage, there was minimal change in stiffness after incubation with hyaluronidase to dissolve glycosaminoglycans, while there was complete loss of mechanical stiffness after incubation with elastase [98]. In summary, both HA and elastin appear to play an important role in the mechanical properties of auricular cartilage with implications for cartilage malleability. The relative importance of elastin versus HA in the context of auricular molding requires further research.

This scoping review also found a lack of sufficient evidence to provide a clinical recommendation for breastfeeding for patients undergoing auricular molding. There were only four primary sources cited among all studies describing the impact of breastfeeding on auricular molding outcome. One source anecdotally described a need for prolonged auricular molding treatment in patients that were breastfed. Among the other three sources, one large case series found that breastfeeding improved molding outcomes, while the other two found no difference in outcomes [33, 45, 79]. In all three studies, there was little information regarding the auricular anomaly type, severity, age at treatment initiation, and duration of treatment between patient groups. In two other longitudinal studies on the impact of breastfeeding on infant estrogen levels, one found no difference in estrogen levels between patients who were fed with breastmilk, soy formula, or cow formula from birth to 1 year of age, and one found no difference in the proportion of samples having detectable levels of estradiol in infants fed with soy formula versus breastmilk [101, 102]. More prospective studies of patients undergoing auricular molding matched by auricular anomaly type and severity are necessary to draw stronger conclusions about the impact of breastfeeding on estrogen levels and molding outcomes.

Overall, the findings from this review regarding auricular molding treatment and outcomes are largely consistent with those from recent systematic reviews. There was no consensus on optimal age at initiation of treatment or optimal treatment window, although most studies suggested that earlier initiation was associated with improved outcomes. The timing of initial patient presentation may hinder early initiation of treatment, and some suggest that the newborn hearing screen may be a prime opportunity for identification of auricular anomalies to initiate molding within 3 days of birth [60]. Similarly, there was no consensus on ideal duration of treatment. The difficulty in standardizing treatment protocols is in part due to high variability in auricular deformations, unstandardized categorization and grading schemes, and the subjective nature of physician evaluation or family survey outcome measures.

## 5 Conclusion

Auricular molding is an effective and increasingly popular treatment for congenital auricular anomalies. While there is no standardization for treatment initiation or treatment duration, there is a general consensus that earlier initiation of molding is associated with improved outcome. There is no clear evidence that estrogen increases HA levels in cartilage. The impact of HA on cartilage malleability also remains unclear; some studies suggest that elastin may play an equal, if not greater role in the elastic stiffness of auricular cartilage, and further research is necessary to elucidate the individual contributions of the cartilage constituents to its mechanical properties. Given the scarcity of high quality evidence, no clinical recommendations can be made regarding breastfeeding and auricular molding outcomes.

## Funding

The authors have nothing to report.

## Conflicts of Interest

The authors declare no conflicts of interest.

## Supporting information


**Supporting Information: Table 1** Database Search Strategy.

**Supporting Information: Table 2** Summary of Included Studies.* *Note that the sum of various auricular anomaly counts do not equal the total number of anomalies due to some studies lacking a breakdown of the anomaly type.

## Data Availability

Data sharing not applicable to this article as no datasets were generated or analyzed during the current study.
