# Supplementary material for: Current Evidence of the Effect of Breastfeeding on Ear Molding Outcomes: A Scoping Review
Source: Laryngoscope. 2026 Feb 6;136(7):2868–75. doi: 10.1002/lary.70413 (PMC13253182; doi:10.1002/lary.70413)
Supplement: Supplementary file 1 — Supporting Information: Table 1 Database Search Strategy. [file LARY-136-2868-s001.docx]

| Database | Search Strategy |
| --- | --- |
| Embase | (“ear” OR “auricle” OR “auricular”) AND (“deformity” OR “abnormality” OR “malformation” OR “microtia”) AND (“mold” OR “molding” OR “splint” OR “splinting” OR “nonsurgical” OR “noninvasive”) |
| MEDLINE | (“ear” OR “auricle” OR “auricular”) AND (“deformity” OR “abnormality” OR “malformation” OR “microtia”) AND (“mold” OR “molding” OR “splint” OR “splinting” OR “nonsurgical” OR “noninvasive”) |
| CENTRAL | (“ear” OR “auricle” OR “auricular”) AND (“deformity” OR “abnormality” OR “malformation” OR “microtia”) AND (“mold” OR “molding” OR “splint” OR “splinting” OR “nonsurgical” OR “noninvasive”) |
