# Supplementary material for: Current Evidence of the Effect of Breastfeeding on Ear Molding Outcomes: A Scoping Review
Source: Laryngoscope. 2026 Feb 6;136(7):2868–75. doi: 10.1002/lary.70413 (PMC13253182; doi:10.1002/lary.70413)
Supplement: Supplementary file 2 — Supporting Information: Table 2 Summary of Included Studies.* *Note that the sum of various auricular anomaly counts do not equal the total number of anomalies due to some studies lacking a breakdown of the anomaly type. [file LARY-136-2868-s002.docx]

| Study | Journal Specialty | Study Design | Malformation and Deformation | Number of Patients/Ears | Age at Treatment Start | Duration of Treatment | Ear Molding Technique | Outcome Measures | Discusses Perinatal Estrogen Effect | Cited Primary Source Regarding Estrogen Effect | Discusses Breastfeeding Effect | Cited Primary Source Regarding Breastfeeding Effect |
| --- | --- | --- | --- | --- | --- | --- | --- | --- | --- | --- | --- | --- |
| Ahmed et al., 2023 | Craniofacial | Retrospective Case Series | 2 lop, 2 cup, 1 conchal crus, 3 constricted, 1 prominent, 3 convex conchae | 9/16 | Mean 17, range 4–23 weeks | Mean 5.7 weeks | EarWell | Family satisfaction and clinical score | Supports | Yes | N/A | N/A |
| Alper et al., 2024 | Plastic Surgery | Retrospective Case Series | 19 lop/lidding, 4 Stahl's, 10 helical, 3 prominent, 2 conchal crus, 8 constricted, 1 cryptotia, 15 mixed | 38/62 | Mean 23.2 (19.7) days | Mean 21.7 (7.7) days | EarWell | Caretaker satisfaction and physician evaluation | Supports | Yes | N/A | N/A |
| Bhatti et al., 2021 | Pediatrics | Literature Review | N/A | N/A | N/A | N/A | N/A | N/A | Neutral | No | N/A | N/A |
| Brown et al., 1986 | Pediatrics | Retrospective Case Series | 2 lop, 1 cup, 1 Stahl's, 1 helical | 4/5 | Either 2 days, 6 days, 4 days, or 12 weeks | Range 3–8 weeks | Dental Compound (Aluwax) with Steri-Strips | Assessment of photograph and physical examination results | Supports | Yes | N/A | N/A |
| Burns et al., 2023 | Plastic Surgery | Literature review | N/A | N/A | N/A | N/A | N/A | N/A | Supports | Yes | N/A | N/A |
| Byrd et al., 2010 | Plastic Surgery | Retrospective Case Series | Unspecified | 34/58 | Unspecified | 6 weeks | EarWell | Classification of auricular morphologic result | Supports | Yes | Supports | No |
| Chan et al., 2019 | Plastic Surgery | Retrospective Case Series | 30 lidding, 19 Stahl's, 10 helical, 5 prominent, 5 lop, 1 conchal crus, 34 constricted, 1 cryptotia | 67/105 | Range 0–7 days | Mean 4.1 weeks | EarWell | Assessment of photograph and physical examination results | Supports | Yes | Rejects | Primary Study |
| Chang and Bartlett, 2017 | Pediatrics | Retrospective Case Series | 8 Stahl's ear, 6 lop, 7 prominent, 10 helical deformity, 2 mixed | 24/33 | Mean 31.21, range 6–106 days | Mean 3.85 weeks, range 1.7–6.57 weeks | Velcro, silicone rubber conformer, polysiloxane gel (Memosil) | Grading on physical examination | Supports | Yes | N/A | N/A |
| Chang and Bartlett, 2019 | Pediatrics | Literature review | N/A | N/A | N/A | N/A | N/A | N/A | Supports | Yes | Supports | No |
| Chen et al., 2021 | Pediatrics | Retrospective Case Series | 20 cryptotia, 50 lop, 111 helical, 25 cup, 27 constricted, 16 Stahl's, 17 mixed | 173/274 | Mean 15 days, range 3 days–3 years | Prior to 14 days, mean = 35.88 (21.87) days, after 14 days, mean = 40.29 (23.66) days | EarWell | Ear length and width | Supports | Yes | N/A | N/A |
| Chen et al., 2023 | Plastic Surgery | Prospective Case Series | Constricted ears with 34.1% mixed, 4.4% cryptotia, 5.5% Stahl's, 11% helical, 13.2% conchal crus | 60/91 | Mean 19.2, range 2–107 days | 6–8 weeks of expansion, then 1–2 weeks of maintenance | EarWell | Assessment of photograph and physical examination results and parent satisfaction scoring | Supports | No | N/A | N/A |
| Chia and Sim, 2021 | Plastic Surgery | Prospective Case Series | Cryptotia | 5/7 | 1 day–7 months | Range 2–4 weeks | EarWell | Assessment of photograph and physical examination results and parent satisfaction rating | Neutral | No | N/A | N/A |
| Dinis et al., 2022 | Plastic Surgery | Retrospective Case Series | 30 lop/lidding, 15 Stahl's, 25 helical, 8 prominent, 6 cup, 2 conchal crus, 11 constricted, 2 cryptotia, 22 mixed | 74/121 | Mean 20.1 (21.4) days | Mean 21.1 (7.7) days | EarWell or InfantEar | Parent satisfaction regarding result/appearance, burden of cleaning/care, physician assessment of aesthetic outcome | Supports | Yes | N/A | N/A |
| Doft et al., 2015 | Plastic Surgery | Prospective Case Series | 38% helical, 25% Stahl's, 18% constricted, 18.5% cryptotia, 0.5% prominent conchal strut | 96/158 | <1 wk: 130, <2 weeks: 20, 2–6 weeks: 8 | Mean 14 days, range 7–42 days) | EarWell | Assessment of photograph and physical examination results | Supports | No | N/A | N/A |
| Duvdevani et al., 2022 | Otolaryngology | Retrospective Case Series | 4 microtia, 10 cup, 14 helical, 9 prominent, 7 Stahl's, 5 lidding, 2 constricted, 1 conchal crus, 1 inverted conchal bowl | 31/54 | Mean 26.8 days | Mean 43.2 days, median 28 days | Silicone custom mold | Physical examination | Neutral | No | N/A | N/A |
| Feijen et al., 2020 | Plastic surgery | Literature Review | N/A | N/A | N/A | N/A | N/A | N/A | Neutral | Yes | Neutral | Yes |
| Hallac et al., 2021 | Other | Translational research | 47.3% lidding, 18% helical, 12% Stahl's, 8.5% constricted, 6.4% prominent, 6% satyr, 1.8% cryptotia | 155/283 | <14 days: 210, >14 days: 73 | <2 weeks: 8  2–4 weeks: 61  4–6 weeks: 61  6–8 weeks: 13  8–10 weeks: 8  10–12 weeks: 4 | Unspecified | Comparison of physician assistant rating versus deep learning assessment | Supports | No | N/A | N/A |
| Hilewitz and Olshinka, 2024 | Craniofacial | Prospective Case Series | Microtia | 5/5 | Mean 2 weeks | Mean 6.6 weeks | EarWell | Parent satisfaction survey | Supports | No | N/A | N/A |
| Huang et al., 2024 | Otolaryngology | Systematic Review | Unspecified | 1248/1860 | Unspecified | Range 14–56 days | N/A | N/A | Supports | No | N/A | N/A |
| Hui et al., 2023 | Otolaryngology | Prospective Case Series | 10 cup, 4 lop, 18 helical, 8 mixed, 3 conchal crus, 3 microtia, 20 cryptotia, 4 Stahl's, 4 prominent | 35/59 | Unspecified | Unspecified | EarWell or InfantEar | Assessment of photograph and physical examination results and treatment decision-maker psychological questionnaire | Supports | Yes | Rejects | Yes |
| Kim et al., 2022 | Other | Systematic Review | 65 lop, 12 constricted, 7 helical, 7 Stahl's, 3 prominent | 54/94 | Mean 5.82 (6.09) days, range 1–22 days | Mean 32.13 (7.90) days, median 35 days, range 15–47 days | Custom-made Splints (metal Wire, surgical Tape, foam, and silicone tape) | Physical examination | Supports | No | N/A | N/A |
| Koshy et al., 2025 | Plastic Surgery | Prospective Case Series | 47% helical, 43% prominent, remainder conchal deformity or other cartilage abnormality | 123/182 | Median 8 weeks, range 1–52 weeks | Median 10 weeks, range 3–36 weeks | EarBuddies | Patient reported pre- and post- splint questionnaires assessing appearance, shape, and prominence of ear. | Neutral | Yes | N/A | N/A |
| Lennon and Chinnadurai, 2018 | Otolaryngology | Literature review | N/A | N/A | N/A | N/A | N/A | N/A | Supports | No | N/A | N/A |
| Leonardi et al., 2012 | Other | Retrospective Case Series | 6 lop, 8 constricted, 4 prominent, 4 Stahl's | 12/22 | Range 2–42 days | Range 5–8 weeks | Soldering wire within suction catheter, Steri-strips | Physician and parent score from 0–10 | Neutral | Yes | N/A | N/A |
| Li et al., 2023 | Pediatrics | Prospective Case Series | 8 Stahl's, 10 helical, 6 cup, 8 lop | 16/32 | Mean 4.37 (1.28) days | Mean 34.62 (6.03) days | Full GTK Ear Correction Kit (one ear), only retractor and antihelix former from EarWell (contralateral ear) | Parent and physician independent evaluation (3 levels of grading) | Neutral | No | N/A | N/A |
| Lindford et al., 2007 | Other | Retrospective Case Series | 2 constricted, 1 Stahl's, 2 prominent | 3/5 | 3 days | Range 3–4 weeks | Wire in 6 Fr silastic tube | N/A | Supports | No | N/A | N/A |
| Liu et al., 2022 | Pediatrics | Retrospective Case Series | 39 prominent, 74 lidding, 111 helical, 13 Stahl's, 7 prominent conchal crus, 8 mixed, 84 constricted | 224/356 | Median 39.5 days | Median 42.5 days | EarWell | Assessment of photographs | Supports | No | Supports | Yes |
| Lv et al., 2024 | Craniofacial | Retrospective Case Series | 26% helical, 13.1% lop, 8.9% cup, 6.5% Stahl's, 2.8% conchal crus, 2.7% prominent, 3.8% constricted, 3.6% cryptotia, 1.9% microtia | 331/527 | Mean 25 (28) days | Mean 7 (5) weeks | EarWell or InfantEar | Assessment of photograph and physical examination results | Neutral | No | Rejects | Yes |
| Matsuo et al., 1984 | Plastic Surgery | Literature Review | N/A | N/A | N/A | N/A | N/A | N/A | Supports | Yes | N/A | N/A |
| Matsutani et al., 2023 | Craniofacial | Retrospective Case Series | 49 cryptotia, 31 constricted | 63/80 | Mean 3.5, range 0–51 months | Mean 3.75, range 0.5–11.5 months | Metal paper clip, cotton balls, thermoplastic resin | Assessment of photograph and physical examination results | Supports | Yes | N/A | N/A |
| Mohammadi et al., 2016 | Plastic surgery | Prospective Case Series | 11 prominent, 8 lop, 10 constricted | 29/29 | Mean 7.52 (5.60) weeks | Mean 13.3 (2.0) weeks | Silicone with stainless steel wire, surgical tape | Assessment of photograph and physical examination results | Supports | Yes | Supports | No |
| Nigam et al., 2020 | Craniofacial | Retrospective Case Series | 96 mixed, 63 prominent, 39 helical, 24 lidding/lop, 23 Stahl's, 21 cup, 6 conchal crus | 175/272 | Mean 20.4 (18) days | Mean 34.8 days for EarWell, mean 32.4 days for InfantEar | EarWell or InfantEar | Comparison of EarWell vs Infant Ear in early (<3 weeks) and delayed (>3 weeks) based on photographic assessment, family satisfaction, and skin complications | Supports | No | N/A | N/A |
| Olshinka et al., 2021 | Craniofacial | Retrospective Case Series | Unspecified | 8/9 | <12 weeks | Mean 10.25 weeks | EarWell, custom silicone ear mold if needed (Azoft) |  | Supports | No | N/A | N/A |
| Olshinka et al., 2022 | Craniofacial | Retrospective Case Series | Curved concha | 10/11 | Mean 2 weeks | Mean 5.2 weeks | EarWell | Clinical asessment and family satisfaction | Supports | No | N/A | N/A |
| Oroz et al., 1995 | Plastic Surgery | Retrospective Case Series | 16 prominent, 14 lop, 9 cup, 8 lobe evertion, 6 Stahl's | 32/45 | <5 days | Range 2–3 weeks | Godiva mold; steel wire in silicon tube | Physical examination | Supports | Yes | N/A | N/A |
| Petersson et al., 2012 | Otolaryngology | Prospective Case Series | 6 Stahl's, 7 cup, 4 prominent | 9/17 | 1–2 days | Range 4 days–13 weeks | Copper wire in 6Fr silicone tube | Assessment of photograph and physical examination results | Neutral | Yes | N/A | N/A |
| Porter et al., 2005 | Plastic Surgery | Literature Review | N/A | N/A | N/A | N/A | N/A | N/A | Supports | No | N/A | N/A |
| Roby et al., 2023 | Otolaryngology | Literature Review | N/A | N/A | N/A | N/A | N/A | N/A | Neutral | No | N/A | N/A |
| Ruder et al., 1996 | Pediatrics | Literature Review | N/A | N/A | N/A | N/A | N/A | N/A | Supports | No | N/A | N/A |
| Saba et al., 2022 | Otolaryngology | Systematic Review | N/A | 1729/2508 | N/A | N/A | N/A | N/A | Supports | No | N/A | N/A |
| Sayadi et al., 2023 | Plastic Surgery | Retrospective Case Series | 50 helical, 40 lop, 7 lidding, 4 antihelix deformity, 13 prominent, 20 Stahl's, 19 constricted, 15 cup, 7 cryptotia, 9 mixed | 114/184 | Mean 21 days | Mean 40 days | EarWell | Degree of ear anomaly correction and parent questionnaire on long-term satisfaction | Supports | Yes | Neutral | No |
| Schonauer et al., 2003 | Plastic Surgery | Retrospective Case Series | 9 constricted, 7 Stahl's, 10 helical, 10 bat | 26/36 | Unspecified | Range 2–6 weeks | Wire core in 6Fr Silastic tube and tape | Physical examination | Supports | No | N/A | N/A |
| Schonauer et al., 2009 | Plastic Surgery | Retrospective Case Series | 37 vertical deformity, 23 horizontal deformity, 7 mixed, 5 helical | 46/72 | 2–3 days | Range 3–6 weeks | Wire core in 6Fr Silastic tube and tape | Physical examination | Supports | Yes | N/A | N/A |
| Schultz et al., 2017 | Plastic Surgery | Literature Review | N/A | N/A | N/A | N/A | N/A | N/A | Supports | No | N/A | N/A |
| Seo et al., 2024 | Plastic Surgery | Prospective Cohort Study | 9 prominent, 24 constricted, 11 helical, 9 other | 35/53 | Early group: median 12 days, IQR 5–14 days  Late group: median 113 days, IQR 94.5–170 days | Early group: median 4 weeks, IQR 4–6 weeks  Late group: median 8 weeks, IQR 4–12 weeks | BabyEar | Aesthetic outcomes, caregiver satisfaction | Supports | Yes | N/A | N/A |
| Sulibhavi et al., 2024 | Otolaryngology | Systematic Review | N/A | 3431 | Median 4.8 weeks, range 0.9–8.8 weeks | Median 4.7, range 2.6–7.6 weeks | N/A | N/A | Supports | No | N/A | N/A |
| Tan et al., 1994 | Plastic Surgery | Retrospective Case Series | 4 Stahl's, 2 lop, 1 shell, 1 kink, 2 prominent | 10/14 | Range 6 hours–5 months | Range 10 days–3 months | Soldering wire in 8Fr suction catheter | Assessment of photograph and physical examination results | Neutral | Yes | N/A | N/A |
| Tan et al., 1997 | Plastic Surgery | Retrospective Case Series | 8 prominent, 23 lop, 2 Stahl's, 1 inverted concha | 19/32 | Mean 16.9 days, range 1 day–10 weeks | Mean 9.1 weeks, range 5–21 weeks | Soldering wire inserted in 8Fr suction catheter | Parent and physician assessment on grading scale, helical-mastoid distance | Supports | Yes | Supports | Primary Study |
| Ullmann et al., 2002 | Plastic Surgery | Prospective Case Series | 28 lop, 20 constricted, 24 prominent, 20 Stahl's | 52/92 | Range 1–10 days | Mean 6.8 weeks, range 6–12 weeks | Putty Soft | Layperson and parent evaluation of photographs | Neutral | Yes | N/A | N/A |
| van Cruchten et al., 2024 | Plastic Surgery | Prospective Case Series | 8 conchal crus, 3 constricted, 14 cup, 19 helical, 15 lop, 21 mixed, 36 prominent, 6 Stahl's | 73/123 | Mean 35.5 (18.9) days, range 7–80 days | Mean 59 (27.6) days, range 27–154 days | EarWell | Parent/physician satisfaction | Supports | Yes | Rejects | Primary Study |
| van Wijk et al., 2009 | Plastic Surgery | Systematic Review | N/A | N/A | N/A | N/A | N/A | N/A | Neutral | Yes | Neutral | Yes |
| van Wijk et al., 2012 | Plastic Surgery | Prospective Case Series | Prominent ears | 132/209 | Mean 8.8 weeks, range 0 week–39 weeks | Minimum 4 weeks | EarBuddies | Assessment of photograph and physical examination results; mastoid-helical distance | Neutral | Yes | N/A | N/A |
| van Wijk et al., 2019 | Plastic Surgery | Literature Review | Prominent ears | N/A | N/A | N/A | N/A | N/A | Neutral | No | N/A | N/A |
| Vella, 2024 | Otolaryngology | Literature Review | N/A | N/A | N/A | N/A | N/A | N/A | Rejects | Yes | Rejects | Yes |
| Wang et al., 2020 | Otolaryngology | Retrospective Case Series | Cryptotia | 35/49 | Median 3.5 months, range 1.5 months–4 years 3 months | Range 1 day–6 months | Silicone gel mold | Pre/posttreatment evaluation by two blinded investigators | Supports | Yes | N/A | N/A |
| Wang et al., 2022 | Otolaryngology | Retrospective Case Series | 21 ringed, 32 cup, 35 droop, 69 cryptotia, 65 helical, and 11 mixed | 156/233 | Mean 53.35 (38.05) days, range 10–180 (one outlier of 330 days) | Range 21–50 days | Beautiful Ear orthotic system | Physical examination | Supports | No | N/A | N/A |
| Woo et al., 2016 | Plastic Surgery | Retrospective Case Series | Constricted (18, 64.2%), Stahl's (6, 21.4%), prominent (2, 7.1%), cryptotia (2, 7.1%) | 18/28 | Mean 22.6 days, range 5–52 days | mean 32.7 days (range, 24–53 days) | EarWell | Parent survey of degree of improvement, level of procedural discomfort | Supports | No | N/A | N/A |
| Woo et al., 2017 | Other | Retrospective Case Series | Satyr (28 ears, 35%), forward-facing ear lobe (23 ears, 28.75%), Darwinian notch (14 ears, 17.5%), overfolded (11 ears, 13.75%), cup (9 ears, 11.25%) | 54/80 | Mean 52.91 (18.26) days, range 21–90 days | Mean 44.27 (32.06) days, range 8–169 days | BabyEars | Physician and caregiver ratings after treatment based on VAS | Neutral | No | N/A | N/A |
| Wu et al., 2022 | Plastic Surgery | Systematic Review | 204 prominent, 153 Stahl's, 128 lop, 286 cryptotia, 173 helical, 52 constricted | Unspecified/1727 | Unspecified | N/A | N/A | N/A | Supports | No | N/A | N/A |
| Wu et al., 2025 | Otolaryngology | Systematic Review | N/A | Unpsecified/1027 | Range 0–180 days | Range 1–8 weeks | EarWell | N/A | Neutral | No | N/A | N/A |
| Wu et al., 2025 | Plastic Surgery | Retrospective Case Series | 40 (8.8%) prominent, 27 (6.0%) Stahl's, 207 (45.7%) helical, 66 (14.6%) cup/lop, 28 (6.2%) conchal bowl, 8 (1.8%) cryptotia, 17 (3.8%) constricted, and 60 (13.2%) mixed | 300/453 | Median 2.3, range 1.5–3.6 weeks | Median 4.6, range 3.8–6.0 weeks | EarWell or InfantEar | Assessment of photograph and physical examination results, parent satisfaction survey, deformity severity survey from laypeople | Supports | No | N/A | N/A |
| Xiong et al., 2021 | Otolaryngology | Retrospective Case Series | Cryptotia, prominent, helical, cup | 462/522 | Range 12–112 days | Range 14–42.6 days | EarWell or LiangEar molding system | Postreatment outcome grading | Supports | No | Supports | Primary Study |
| Xu et al., 2023 | Otolaryngology | Retrospective Case Series | Cryptotia | 51/? | Range 6 months–14 years old | Mean 38 (18.0) days in 6 months–1 year group, mean 72 (25.7) days in 1–3 years group, mean 90 (50.6) days in 3–6 years group, and mean 127 (48.5) days in ≥6 years group | EarWell | Effective rate and recurrence rate, and treatment duration of non-surgical ear molding correction compared between age groups | Supports | No | N/A | N/A |
| Zhang et al., 2019 | Otolaryngology | Retrospective Case Series | 24 cryptotia, 29 lidding, 31 cup, 41 helical, 1 Stahl's, 15 mixed | 105/141 | Range 0.23–12.0 months | Range 0.33–4.0 months | EarWell | Treatment age, treatment time and efficiency | Supports | No | N/A | N/A |
| Zhorov et al., 2025 | Otolaryngology | Literature Review | N/A | N/A | N/A | N/A | N/A | N/A | Supports | Yes | Supports | No |
| Zhu et al., 2021 | Otolaryngology | Retrospective Case Series | 11 constricted, 2 Stahl's, 3 lop | 12/16 | Range 0.6–7 months | Range 1–28 weeks | 3D-printed correction brackets | Assessment of photograph and physical examination results | Supports | No | N/A | N/A |
| Zhuang et al., 2020 | Plastic Surgery | Retrospective Case Series | Lop, helical, conchal crus, Stahl's, prominent, cup, cryptotia, constricted | 100/129 | Mean 35.13 (22.75) days | Mean 17.81 (13.01) days | Earlimn | Assessment of photograph and physical examination results | Supports | Yes | N/A | N/A |
